# Supplementary material for: Multi‐dataset identification of innovative feature genes and molecular mechanisms in keratoconus
Source: J Cell Mol Med. 2024 Sep 19;28(18):e70079. doi: 10.1111/jcmm.70079 (PMC11412914; doi:10.1111/jcmm.70079)
Supplement: Supplementary file 2 — Material S2. [file JCMM-28-e70079-s002.zip › 7.GSEA analysis/5.RNF217-AS1/KEGG_TERPENOID_BACKBONE_BIOSYNTHESIS.html]

Details for gene set KEGG\_TERPENOID\_BACKBONE\_BIOSYNTHESIS[GSEA]

|  || Dataset | RNF217-AS1.group.cls#h\_versus\_l |
| Phenotype | group.cls#h\_versus\_l |
| Upregulated in class | h |
| GeneSet | KEGG\_TERPENOID\_BACKBONE\_BIOSYNTHESIS |
| Enrichment Score (ES) | 0.41409528 |
| Normalized Enrichment Score (NES) | 1.049681 |
| Nominal p-value | 0.44129553 |
| FDR q-value | 1.0 |
| FWER p-Value | 0.998 |
Table: GSEA Results Summary

  

Fig 1: Enrichment plot: KEGG\_TERPENOID\_BACKBONE\_BIOSYNTHESIS      
 Profile of the Running ES Score & Positions of GeneSet Members on the Rank Ordered List

  

| SYMBOL | TITLE | RANK IN GENE LIST | RANK METRIC SCORE | RUNNING ES | CORE ENRICHMENT || 1 | IDI2 | na | 568 | 0.516 | 0.4141 | Yes |
| 2 | HMGCS2 | na | 9330 | 0.099 | 0.2441 | No |
| 3 | MVD | na | 9589 | 0.094 | 0.3147 | No |
| 4 | IDI1 | na | 11772 | 0.058 | 0.3006 | No |
| 5 | FDPS | na | 12266 | 0.052 | 0.3297 | No |
| 6 | HMGCR | na | 13118 | 0.041 | 0.3391 | No |
| 7 | MVK | na | 13552 | 0.036 | 0.3563 | No |
| 8 | PDSS1 | na | 13680 | 0.034 | 0.3810 | No |
| 9 | ACAT2 | na | 17362 | -0.005 | 0.2793 | No |
| 10 | PMVK | na | 19141 | -0.021 | 0.2458 | No |
| 11 | DHDDS | na | 19325 | -0.023 | 0.2601 | No |
| 12 | HMGCS1 | na | 19419 | -0.024 | 0.2778 | No |
| 13 | ACAT1 | na | 22576 | -0.057 | 0.2344 | No |
| 14 | PDSS2 | na | 23370 | -0.066 | 0.2671 | No |
| 15 | GGPS1 | na | 23778 | -0.072 | 0.3154 | No |
Table: GSEA details [plain text format]

  

Fig 2: KEGG\_TERPENOID\_BACKBONE\_BIOSYNTHESIS      
 Blue-Pink O' Gram in the Space of the Analyzed GeneSet

  

Fig 3: KEGG\_TERPENOID\_BACKBONE\_BIOSYNTHESIS: Random ES distribution      
 Gene set null distribution of ES for **KEGG\_TERPENOID\_BACKBONE\_BIOSYNTHESIS**
